# Determinants of exercise capacity in heart failure with preserved ejection fraction: central hemodynamics, ventilatory efficiency, and peripheral muscle oxygen utilization and function

**DOI:** 10.3389/fcvm.2026.1854913

**Published:** 2026-07-15

**Authors:** Raphael Schoch, Julia Maria Kröpfl, Benedikt Andreas Gasser, Denis Infanger, Henner Hanssen, Rupprecht Wick, Luisa Prechtl, Thomas Dieterle, Arno Schmidt-Trucksäss

**Affiliations:** 1 Department of Sport, Exercise and Health, Division Sports and Exercise Medicine, University of Basel, Basel, Switzerland; 2 Clinic Arlesheim, Division of Cardiology, Arlesheim, Switzerland; 3 Department of Clinical Research, University Hospital Basel, Basel, Switzerland

**Keywords:** cardiac function, exercise intolerance, heart failure with preserved ejection fraction, peripheral muscle function, ventilatory efficiency

## Abstract

**Background:**

Exercise intolerance, reflected by reduced peak oxygen uptake (V˙O_2_peak), is a hallmark feature of heart failure with preserved ejection fraction (HFpEF). Impairments along the oxygen cascade, including central hemodynamics, ventilatory efficiency, and peripheral factors, have been described, but their relative contributions remain incompletely defined.

**Methods:**

This cross-sectional analysis included baseline data from the HIT-HF trial. V˙O_2_peak and ventilatory efficiency were assessed by cardiopulmonary exercise testing. Stroke volume was measured using impedance cardiography, heart rate via electrocardiography, total hemoglobin mass (tHbmass) by carbon monoxide rebreathing, muscle oxygen saturation (SmO_2_) by near-infrared spectroscopy, and leg power by countermovement jump testing. Associations with relative and absolute V˙O_2_peak were examined using multivariable linear regression adjusted for age, sex, and body fat mass. Missing data were handled by multiple imputations.

**Results:**

Of 46 patients, 43 were included in the final analysis [mean age: 73 (10) years, BMI: 25.6 (4.3) kg/m^2^, 65% female]. In multivariable models, leg power showed the strongest positive association with relative V˙O_2_peak (*β* = 0.35, 95% CI: −0.20 to 0.89), whereas leg fat-free mass showed the strongest association with absolute V˙O_2_peak (*β* = 0.57, 95% CI: 0.21–0.92). Stroke volume and heart rate showed smaller associations, whereas SmO_2_, tHbmass, and ventilatory efficiency showed trivial associations (*β* between −0.1 and 0.1).

**Conclusion:**

In HFpEF, peripheral muscle function was more strongly associated with exercise capacity than central hemodynamics or ventilatory efficiency.

## 1 Introduction

The prevalence of heart failure with preserved ejection fraction (HFpEF) continues to rise, driven by aging populations and increasing rates of obesity and cardiometabolic disease (1). A hallmark feature characterizing this syndrome is reduced exercise tolerance, which reflects a complex interplay of impairments in central hemodynamic, pulmonary, and peripheral muscle oxygen utilization and function (2, 3).

Exercise training improves exercise capacity in patients with HFpEF (4, 5) and is recommended as part of clinical management (6). However, despite these benefits, exercise intolerance remains a hallmark of this syndrome. The physiological mechanisms limiting peak oxygen uptake (V˙O_2_peak), and thereby constraining the response to exercise, are not fully understood. A more comprehensive characterization of the interplay between central and peripheral factors is therefore essential to better explain exercise limitation in HFpEF (7–9).

Several studies have examined central and peripheral determinants of exercise capacity and demonstrated that both central and peripheral mechanisms play a substantial role in reducing exercise tolerance in HFpEF. From a physiological perspective, exercise capacity can be described by the Fick principle, whereby V˙O_2_peak is determined by the product of cardiac output and arteriovenous oxygen difference (a-vDO_2_). Accordingly, limitations in either the central hemodynamic response (stroke volume and heart rate) or peripheral factors related to skeletal muscle perfusion, oxygen extraction, and metabolic utilization can independently or in combination limit exercise capacity (10). Borlaug (9) emphasized that exercise intolerance in HFpEF reflects an integrated limitation of cardiac, vascular, and peripheral reserve rather than an isolated abnormality, underscoring the need to consider multiple interacting mechanisms when evaluating impaired exercise capacity.

Bhella et al. (11) demonstrated that several indices of cardiac reserve were preserved in well-compensated patients with HFpEF despite reduced V˙O_2_peak. The hemodynamic response to exercise suggested impaired skeletal muscle oxidative metabolism, characterized by a disproportionate increase in cardiac output relative to oxygen uptake, which may contribute to premature muscle fatigue and inefficient circulatory response that further limit functional capacity.

Haykowsky et al. (12) demonstrated that impaired peripheral oxygen extraction, reflected by a-vDO_2_, is a key determinant of reduced exercise tolerance in HFpEF and may represent a stronger limitation than central cardiac output. This concept was further supported by Dhakal et al. (13) and Tucker et al. (14), who identified abnormalities in peripheral oxygen extraction as major contributors to impaired exercise capacity in HFpEF.

Early evidence for a peripheral contribution was provided by Kitzman et al. (15), who demonstrated that patients with HFpEF exhibit skeletal muscle abnormalities, including a reduced proportion of oxidative type I fibers and a lower capillary-to-fiber ratio, both of which were independently associated with reduced V˙O_2_peak. Extending these findings, Haykowsky et al. (16) showed that adverse skeletal muscle composition, particularly increased intermuscular fat infiltration, is independently associated with reduced V˙O_2_peak.

Beyond these microstructural alterations, changes in overall muscle quantity and quality have been linked to exercise limitation in HFpEF. Haykowsky et al. (17) reported that lower lean body mass is associated with decreased V˙O_2_peak, while Bekfani et al. (18) demonstrated that patients with HFpEF and sarcopenia exhibit significantly lower exercise capacity and quality of life, highlighting the role of muscle mass and strength. Similarly, reduced muscle quality has been linked to decreased V˙O_2_peak (19). Additionally, Cipriano et al. (20) linked higher adiposity and lower lean mass to impaired oxygen uptake kinetics and reduced exercise tolerance, supporting the notion that body composition adversely affects peripheral oxygen diffusion and utilization dynamics.

At the cellular level, Molina et al. (21) provided evidence for impaired skeletal muscle oxidative capacity in HFpEF, demonstrating reduced mitochondrial content, oxidative enzyme activity, and mitofusion-2-expression, all of which were associated with reduced V˙O_2_peak. Furthermore, Naito et al. (22) demonstrated that anemia further exacerbates exercise intolerance in HFpEF by limiting arterial oxygen delivery and impairing peripheral oxygen extraction during exercise, despite preserved cardiac output. Thereby highlighting the contribution of hematological and metabolic factors to peripheral limitations.

Integrating evidence across physiological, structural, cellular, and hematological levels, Haykowsky et al. (23) and Upadhya et al. (24) emphasized that exercise intolerance in HFpEF cannot be explained by cardiac dysfunction alone, but rather reflects a complex interplay of central and peripheral abnormalities. More recently, Pandey et al. (2) reinforced this paradigm by framing HFpEF as a systemic, multisystem disorder in which extracardiac factors, including skeletal muscle dysfunction, body composition, anemia, and aging-related processes, play a central role in limiting exercise capacity. In summary, these findings indicate that exercise intolerance in HFpEF is multifactorial, with peripheral impairments often predominating.

Although prior studies have examined selected central and peripheral determinants of exercise intolerance in HFpEF, an integrated evaluation simultaneously assessing central hemodynamics, ventilatory efficiency, peripheral oxygen delivery, and skeletal muscle function remains limited. To better clarify the relative contribution of these domains to exercise limitation, this study aimed to evaluate the influence of central hemodynamics (cardiac output), ventilatory efficiency (V˙E/V˙CO_2_), peripheral oxygen delivery and utilization [muscle oxygen saturation (SmO_2_), a-vDO_2_, hemoglobin concentration (Hb), and total hemoglobin mass (tHbmass)], and skeletal muscle function (leg power, leg force, and leg fat-free mass) on V˙O_2_peak in patients with HFpEF. These domains were assessed using clinically applicable measurement approaches, enabling an integrated translational characterization of exercise limitation in HFpEF.

## 2 Methods

### 2.1 Study design and patients

This is a cross-sectional analysis of baseline data from the HIT-HF-trial, a randomized controlled study described in detail elsewhere (25). The trial was conducted at the Department of Sport, Exercise and Health at the University of Basel (Basel, Switzerland) and the Division of Cardiology at the Clinic Arlesheim (Arlesheim, Switzerland) from October 2020 to February 2023. For the present analysis, only baseline data collected prior to randomization were used.

Patients were recruited from the Clinic Arlesheim. All participants met the inclusion criteria and were classified as at least NYHA functional class II (25). Due to recruitment challenges during the COVID-19 pandemic, patients who improved to NYHA class I between screening and baseline assessments under standardized medical therapy were also considered eligible. This approach aligns with current guideline recommendations, which endorse exercise training in patients with stable heart failure across NYHA classes I–II (26, 27). Written informed consent was obtained from all patients prior to baseline assessments.

### 2.2 Cardiopulmonary exercise testing (CPET)

V˙O_2_peak was assessed using incremental cycle ergometry (Ergoselect 200, Ergoline, Bitz, Germany), with breath-by-breath gas exchange analysis (MetaMax 3B, Cortex Biophysik GmbH, Leipzig, Germany). Patients performed one of two ramp protocols depending on baseline fitness. Both began with 3 min of rest and 3 min of warm-up (unloaded or 10 W), followed by a continuous workload increase (7 or 10 W/min) until exhaustion, and ended with a 3-min cool-down at 10 W. A consistent pedaling rate of 60 repetitions per minute was requested throughout the protocol and maintained until test termination due to exhaustion or clinical indications. V˙O_2_peak was defined as the highest 30-s averaged oxygen uptake during the test (28).

### 2.3 Central hemodynamics

Stroke volume was measured continuously throughout the CPET using a non-invasive impedance cardiography system (PhysioFlow®, Manatec Biomedical, Poissy, France). This method estimates stroke volume index based on changes in transthoracic impedance during systolic ejection. Six electrodes were placed: two for single-lead electrocardiography (V1/V6) and four for impedance measurement, pairwise on the left side of the neck (between the earlobe and clavicle) and on the thorax near the xiphoid process. The skin at electrode sites was shaved, disinfected, and gently abraded to optimize signal quality. A resting calibration of over 30 cardiac cycles was performed to ensure adequate signal quality. Signal quality was continuously monitored, and only recordings with ≥ 95% signal integrity were included. Cardiac output was calculated as the product of stroke volume and heart rate, both obtained by impedance cardiography. For analysis at V˙O_2_peak, stroke volume values were averaged over a 30-s window centered on V˙O_2_peak. The method has previously been described in more detail (29, 30).

Heart rate was also monitored independently during CPET via a 12-lead electrocardiograph (Custo Med GmbH, Ottobrunn, Germany), and these values were used for further analyses.

### 2.4 Ventilatory efficiency

Ventilatory efficiency was assessed by the V˙E/V˙CO_2_ slope, which was derived via linear regression analysis of ventilation and carbon dioxide output during CPET, excluding non-linear data points near peak exertion (31).

### 2.5 Peripheral oxygen delivery and utilization

SmO_2_, an indicator of the balance between local oxygen delivery and utilization, was assessed using near-infrared spectroscopy (PortaMon, Artinis Medical Systems, Arnhem, Netherlands), which is considered a reasonable approach for assessing skeletal muscle oxygen saturation (32–34). The dual-wavelength device (765 and 848 nm) estimates tissue saturation index (TSI = O_2_Hb/(O_2_Hb + HHb) × 100) with a penetration depth of 15–20 mm, based on the modified Beer-Lambert law. Data were continuously recorded at 10 Hz using Oxysoft software (Version 3.0.103.3, Artinis Medical Systems). During CPET, the sensor was positioned on the left vastus lateralis muscle, one of the most metabolically active muscles while cycling (35), approximately one-third of the distance from the patella to the greater trochanter, aligned parallel to the femur. To minimize motion artefacts, the sensor was secured with medical adhesive tape (Transpore, 3M, Switzerland).

THbmass was determined using the Detalo Performance^TM^ device (Detalo Health ApS, Birkerød, Denmark) based on a standardized carbon monoxide rebreathing protocol. Patients first rested for 10 min in the supine position prior to testing. Subsequently, two capillary blood samples were obtained from the fingertip to determine baseline carboxyhemoglobin (%HbCO) and [Hb] using a blood gas analyzer (RapidPoint 500e, Siemens Healthineers, Erlangen, Germany). Patients then inhaled 100% oxygen for 1 min in a closed breathing circle, followed by rebreathing a bodyweight-adjusted bolus of carbon monoxide (0.75–1.50 mL/kg) mixed with oxygen for 6 min. After a 3-min equilibration phase, another two capillary blood samples were collected to reassess %HbCO and [Hb]. THbmass was calculated based on the change in %HbCO and the amount of carbon monoxide remaining in the rebreathing circle.

A-vDO_2_ was calculated according to the Fick principle (V˙O_2_peak = heart rate * stroke volume * a-vDO_2_).

### 2.6 Peripheral muscle function

Lower extremity muscle power and force were assessed using a countermovement jump (CMJ) performed on the force plate (Leonardo Mechanograph, Novotec Medical, Pforzheim, Germany). Participants were instructed to jump as high as possible while keeping their hands on their hips. For individuals unable to jump, a rapid and forceful push-off was performed to estimate power output. After a 10-min warm-up phase and two familiarization jumps, the test was performed three times with 60-s rest intervals between attempts. Peak leg power (kW) and peak leg force (kN), derived from the vertical ground reaction force, represented the main outcome of the CMJ. This method has shown high reliability in previous studies (36, 37).

Body fat mass and leg fat-free mass were measured using the InBody 720 device (InBody Co. Ltd., Seoul, South Korea) based on four-segment bioelectrical impedance analysis.

### 2.7 Statistical analysis

Descriptive statistics were calculated for all variables and are presented as mean (standard deviation) or median (interquartile range) for continuous variables, and as number (%) for categorical variables.

Multiple linear regression analyses were performed to assess the association between potential predictors and V˙O_2_peak. Model selection was performed by comparing linear and nonlinear models using restricted cubic splines (3 knots) for continuous predictors. Model fit was primarily evaluated using the Akaike Information Criterion (AIC), while partial *F*-tests were used as complementary measures to assess whether increased model complexity significantly improved model fit. Multicollinearity was quantified using the variance inflation factors (VIF), with values <5 considered acceptable (38). Model assumptions were assessed using residual diagnostics, including evaluation of linearity, homoscedasticity, and normality of residuals. Influential observations were examined using standard influence measures, including Cook's distance, leverage, DFFITS, and DFBETAS. Missing data were addressed using multiple imputations by chained equations, based on 100 imputed datasets, including all predictors in the imputation model. The final regression model was fitted separately across all imputed datasets, and estimates were pooled using the Rubin's Rules.

The relative importance of predictors was quantified using the Lindeman-Merenda-Gold (LMG) method, which decomposes the model *R*^2^ into additive contributions of each predictor while accounting for shared explained variance (39).

All statistical analyses were conducted using R software (version 4.3.1, R Foundation for Statistical Computing, Vienna, Austria), multiple imputation using the package “mice” (version 3.16.0), and relative importance using “relaimpo” (version 2.2.7). A significance level of 0.05 was used for all analyses.

## 3 Results

A total of 46 patients with HFpEF were enrolled. Baseline characteristics are summarized in Table 1. The cohort was predominantly female (67%), with a mean age of 73 (9) years, a BMI of 26.1 (5.3) kg/m^2^, and most patients were classified as NYHA class II (46%). Patients had a preserved left ventricular ejection fraction of 63.6 (6.5)%; hypertension was common (72%), and atrial fibrillation was the most frequent comorbidity (28%). The most frequently used medication was an ACE inhibitor or angiotensin receptor blocker (ACE/ARB) (59%).

**Table 1:** Baseline characteristics of patients with HFpEF.

Variables		HFpEF (*n* = 46)	
Age (years), *mean (SD)*		73	(9)
Sex, *no. (%)*	Female	31	(67%)
	Male	15	(33%)
Body mass index (kg/m^2^), *mean (SD)*		26.1	(5.3)
Heart rate (bpm), *mean (SD)*		64	(10)
Blood pressure (mmHg), *mean (SD)*	Systolic	141	(22)
	Diastolic	86	(11)
NT-proBNP (pg/mL), *median (IQR)*		241	(191–300)
NYHA class, *no. (%)*	I	13	(28%)
	II	21	(46%)
	III	12	(26%)
Echocardiography, *mean (SD)*			
Left ventricular ejection fraction (%)		63.6	(6.5)
LAVol (mL/m^2^)		40.1	(12.3)
LVMass (g/m^2^)		93.8	(26.4)
*e*′ septal (cm/s)		8.2	(10.4)
*e*′ lateral (cm/s)		9.7	(6.1)
Mitral valve E velocity (m/s)		0.8	(0.2)
*E*/*e*′ mean		10.1	(3.4)
*E*/*e*′ septal		12.1	(4.4)
*E*/*e*′ lateral		9.0	(3.3)
Cardiovascular risk factors, *no. (%)*			
Hypertension		33	(72%)
Hyperlipidemia		16	(35%)
Diabetes		2	(4%)
Never smoked		26	(57%)
Ex-Smoker		16	(35%)
Current Smoker		4	(9%)
Comorbidities, *no. (%)*			
Coronary artery disease		11	(24%)
Atrial fibrillation		13	(28%)
COPD		4	(9%)
Renal failure		3	(7%)
Stroke		2	(4%)
Medication, *no. (%)*			
ACE/ARB		27	(59%)
Other antihypertensive medication		13	(28%)
*β*-Blockers		9	(20%)
Diuretics		14	(31%)
Cholesterol-lowering drugs		13	(28%)
Antiplatelet		9	(20%)
Quality of life, *mean (SD)*			
KCCQ		72	(20)
MLHFQ		22	(18)

HFpEF, heart failure with preserved ejection fraction; SD, standard deviation; IQR, interquartile range; NT-proBNP, B-type natriuretic peptide; NYHA, New York Heart Association; LAVol, left atrial volume indexed to body surface area; LVMass, left ventricular mass indexed to body surface area; COPD, chronic obstructive pulmonary disease; ACE, angiotensin-converting enzyme inhibitor; ARB, angiotensin receptor blocker; KCCQ, Kansas City Cardiomyopathy Questionnaire; MLHFQ, Minnesota Living with Heart Failure Questionnaire.

Three patients were excluded from further analyses due to missing V˙O_2_peak values, resulting in a final sample size of 43 patients for multivariable regression analyses of exercise capacity. Determinants of exercise capacity are presented in Table 2. The mean relative V˙O_2_peak was 22.0 (5.1) mL/kg/min, and the mean absolute V˙O_2_peak was 1,565 (501) mL/min.

**Table 2:** Determinants of exercise capacity.

Variables		*n*	%
Sex	Female	28	(65%)
	Male	15	(35%)
Variables		Mean	(SD)
Age (y)		73	(10)
Body mass index (kg/m^2^)		25.6	(4.3)
Body fat mass (kg)		23.3	(9.6)
Fat free mass legs (kg)		14.8	(3.6)
[Hb] (g*dL^−1^)		13.47	(1.28)
Total hemoglobin mass (g)		683	(205)
Peak exercise		Mean	(SD)
Heart rate (bpm)		142	(18)
Stroke volume (mL)		103.9	(20.5)
Stroke volume index (mL/m^2^)		57.4	(12.7)
Cardiac output (L/min)		14.5	(3.4)
Cardiac index (L/min/m^2^)		8.0	(2.0)
SmO_2_ (%)		62	(8)
AvDO_2_ (mL/100 mL)		10.9	(3.3)
V˙E/V˙CO_2_		37.8	(5.9)
V˙O_2_peak (mL/kg/min)		22.0	(5.1)
V˙O_2_peak (mL/min)		1,565.31	(500.55)
Strength test		Mean	(SD)
Leg Power (kW)		1.63	(0.72)
Leg Force (kN)		1.34	(0.37)

SD, standard deviation; [Hb], hemoglobin concentration; SmO_2_, muscle oxygen saturation; avDO_2_, arteriovenous oxygen difference; V˙E/V˙CO_2_, ratio of minute ventilation to carbon dioxide output slope; V˙O2peak, peak oxygen uptake.

Comparison of linear and nonlinear models using restricted cubic splines (3 knots) for continuous predictors showed no evidence of improved model fit for the spline-based models (Supplementary Table S1). Therefore, linear terms were used in the final model. To avoid multicollinearity (VIF > 5), [Hb], cardiac output, and leg force were excluded, as their inclusion increased collinearity among predictors. Furthermore, a-vDO_2_ was not included, as it was not an independent measurement but derived from V˙O_2_peak, heart rate, and stroke volume according to the Fick principle. Model assumptions were assessed using residual plots and diagnostic tests. No major violations were detected (Supplementary Figure S1).

The results of the multiple linear regression analyses are summarized in Table 3 (relative V˙O_2_peak) and Table 4 (absolute V˙O_2_peak), presenting standardized regression coefficients. The model explained 62% of the variance in relative V˙O_2_peak (*R*^2^ = 0.62; 95% CI: 0.40–0.78; *n* = 43), and 81% of the variance in absolute V˙O_2_peak (*R*^2^ = 0.81; 95% CI: 0.67–0.89; *n* = 43). Corresponding unstandardized regression coefficients are presented in (Supplementary Table S2 for relative and Supplementary Table S3 for absolute V˙O_2_peak). Sensitivity analyses excluding patients with NYHA class I demonstrated a similar pattern of associations for both the relative and absolute V˙O_2_peak models (Supplementary Tables S4 and S5).

**Table 3:** Multiple linear regression predicting relative V˙O_2_peak (mL/kg/min).

Predictor	*β* (std.)	SE	95% CI	*p* value	LMG
(Intercept)	0.15	0.70	(−1.28; 1.58)	0.784	—
Body fat mass (kg)	−0.73	0.17	(−1.07; −0.40)	< 0.001	41.3%
Stroke volume (mL)	0.24	0.12	(−0.00; 0.49)	0.054	6.0%
Heart rate (bpm)	0.25	0.15	(−0.05; 0.55)	0.097	11.1%
Leg Power (kW)	0.35	0.26	(−0.20; 0.89)	0.200	12.9%
Leg fat-free mass (kg)	0.22	0.24	(−0.28; 0.71)	0.379	4.7%
V˙E/V˙CO_2_	0.07	0.13	(−0.20; 0.35)	0.601	1.2%
SmO_2_	0.06	0.15	(−0.25; 0.37)	0.682	5.0%
Age	−0.07	0.23	(−0.55; 0.40)	0.754	10.8%
Sex (male)	−0.11	0.51	(−1.16; 0.93)	0.826	4.9%
Total hemoglobin mass (g)	0.03	0.22	(−0.43; 0.48)	0.903	2.1%

LMG values indicate the relative contribution of each predictor to the explained variance (*R*^2^) of the model.

V˙O_2_peak, peak oxygen uptake; *β* (std.), standardized regression coefficient; SE, standard error; 95% CI, 95% confidence interval; LMG, Lindeman-Merenda-Gold; SmO_2_, muscle oxygen saturation; V˙E/V˙CO_2_, ratio of minute ventilation to carbon dioxide output slope.

**Table 4:** Multiple linear regression predicting absolute V˙O_2_peak (mL/min).

Predictor	*β* (std.)	SE	95% CI	*p* value	LMG
(Intercept)	−0.01	0.50	(−1.03; 1.01)	0.986	—
Leg fat-free mass (kg)	0.57	0.17	(0.21; 0.92)	0.003	28.9%
Stroke volume (mL)	0.18	0.09	(−0.00; 0.35)	0.050	5.0%
Heart rate (bpm)	0.19	0.10	(−0.02; 0.40)	0.080	6.6%
Leg Power (kW)	0.29	0.19	(−0.09; 0.68)	0.130	21.8%
Body fat mass (kg)	−0.10	0.12	(−0.35; 0.14)	0.385	2.8%
V˙E/V˙CO_2_	0.05	0.10	(−0.15; 0.24)	0.627	0.5%
Age	−0.03	0.17	(−0.37; 0.31)	0.873	12.5%
Total hemoglobin mass (g)	0.02	0.15	(−0.30; 0.35)	0.886	5.6%
SmO_2_	0.01	0.11	(−0.22; 0.23)	0.965	1.4%
Sex (male)	0.01	0.36	(−0.74; 0.76)	0.986	15.0%

LMG values indicate the relative contribution of each predictor to the explained variance (*R*^2^) of the model.

V˙O_2_peak, peak oxygen uptake; *β* (std.), standardized regression coefficient; SE, standard error; 95% CI, 95% confidence interval; LMG, Lindeman-Merenda-Gold; SmO_2_, muscle oxygen saturation; V˙E/V˙CO_2_, ratio of minute ventilation to carbon dioxide output slope.

**Figure 1:**
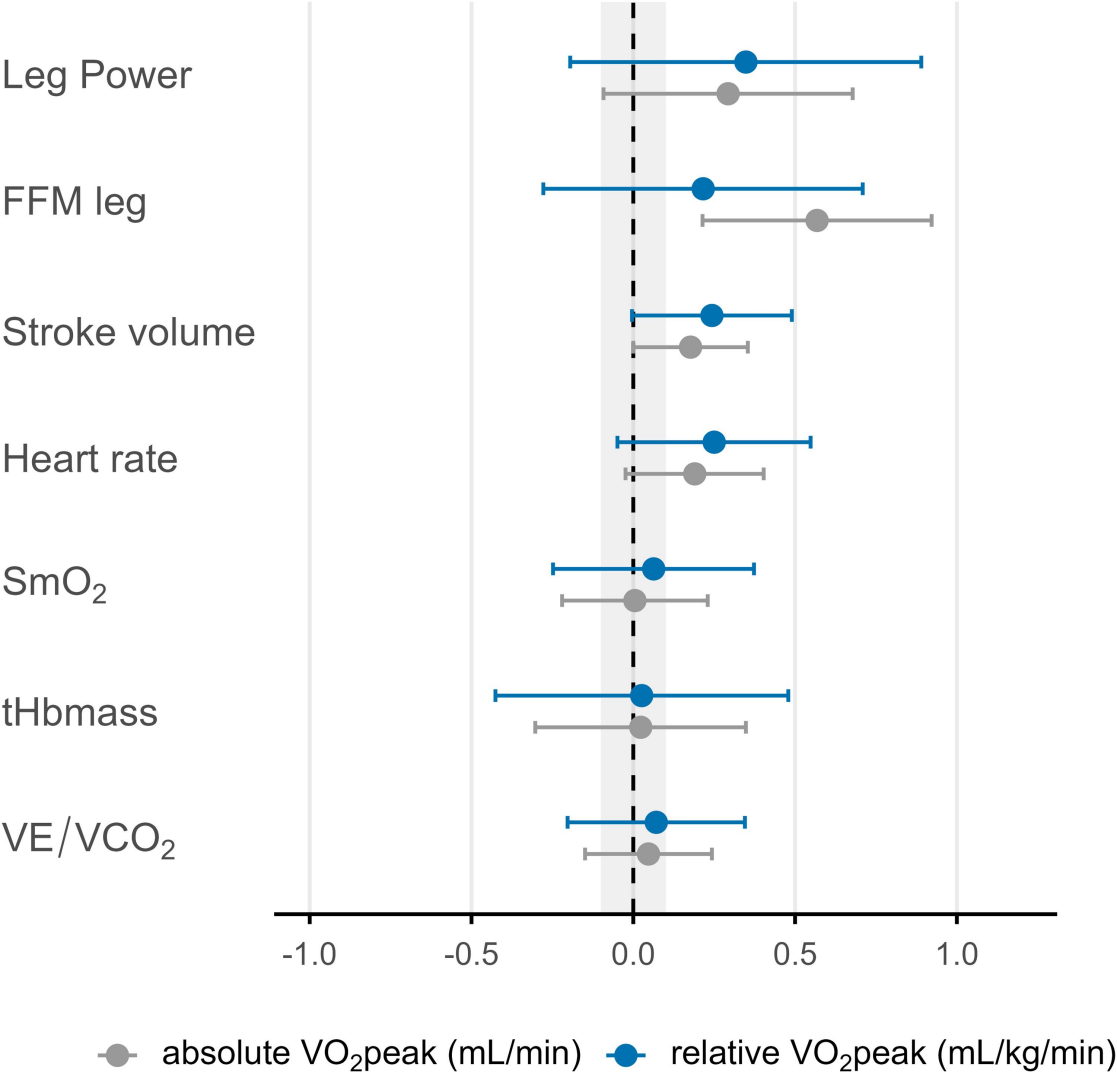
Forest plot of standardized regression coefficients for determinants of absolute (grey) and relative (blue) V˙O_2_peak, based on imputed data, adjusted for age, sex, and body fat mass. Points represent mean standardized regression coefficients; horizontal bars indicate 95% confidence intervals. The grey-shaded area (−0.1 to 0.1) denotes a range interpreted as a trivial effect size. FFM leg, leg fat-free mass; SmO_2_, muscle oxygen saturation; tHbmass, total hemoglobin mass; V˙E/V˙CO_2_, ratio of minute ventilation to carbon dioxide output slope; V˙O_2_peak, peak oxygen uptake.

To visualize the association between determinants and exercise capacity, a forest plot based on imputed data was generated, including both relative and absolute V˙O_2_peak (Figure 1). All estimates were adjusted for age, sex, and body fat mass. Among the included variables, leg power showed the strongest positive association with relative V˙O_2_peak (*β* = 0.35; 95% CI: −0.20 to 0.89; LMG = 12.9%; *p* = 0.200), while stroke volume (*β* = 0.24; 95% CI: −0.00 to 0.49; LMG = 6.0%; *p* = 0.054), heart rate (*β* = 0.25; 95% CI: −0.05 to 0.55; LMG = 11.1%; *p* = 0.097), and leg fat-free mass (*β* = 0.22; 95% CI: −0.28 to 0.71; LMG = 4.7%; *p* = 0.379) demonstrated small effect sizes. In contrast, SmO_2_, tHbmass, and V˙E/V˙CO_2_ were associated with trivial effects (*β* values ranging from −0.1 to 0.1).

A different pattern was observed for absolute V˙O_2_peak, where leg fat-free mass showed the strongest association (*β* = 0.57; 95% CI: 0.21–0.92; LMG = 28.9%; *p* = 0.003). Leg power showed a moderate association (*β* = 0.29; 95% CI: −0.09 to 0.68; LMG = 21.8%; *p* = 0.130), while stroke volume (*β* = 0.18; 95% CI: 0.00–0.35; LMG = 5.0%; *p* = 0.050) and heart rate (*β* = 0.19; 95% CI: −0.02 to 0.40; LMG = 6.6%; *p* = 0.080) showed small effect sizes. SmO_2_, tHbmass, and V˙E/V˙CO_2_ again demonstrated trivial contributions (*β* values ranging from −0.1 to 0.1).

**Figure 2:**
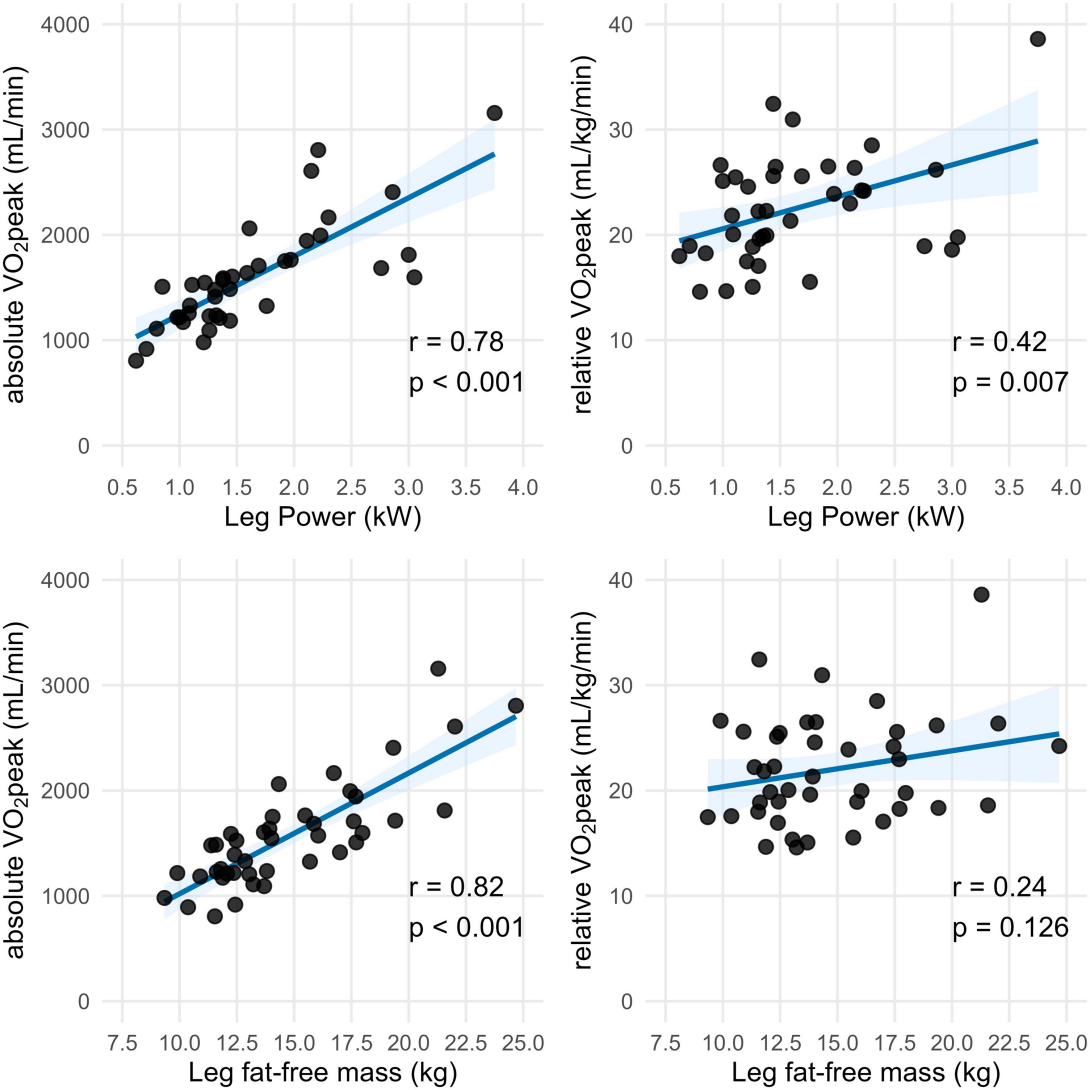
Linear relationships between leg power and leg fat-free mass with peak oxygen uptake (V˙O_2_peak), based on the original, unadjusted dataset. The solid lines represents the linear regression, and the shaded area indicates the 95% CI (leg power vs. absolute V˙O_2_peak: *r* = 0.78; 95% CI: 0.62 to 0.88; *p* < 0.001, leg power vs. relative V˙O_2_peak: *r* = 0.42; 95% CI: 0.12 to 0.65; *p* = 0.007, leg fat-free mass vs. absolute V˙O_2_peak: *r* = 0.82; 95% CI: 0.68 to 0.90; *p* < 0.001, leg fat-free mass vs. relative V˙O_2_peak: *r* = 0.24; 95% CI: −0.07 to 0.50; *p* = 0.126). V˙O_2_peak, peak oxygen uptake.

A corresponding plot based on the original (non-imputed) dataset is provided in Supplementary Figure S2. To further illustrate the strength of the associations between leg power, leg fat-free mass and V˙O_2_peak, linear regression analyses were performed using the original, unadjusted data (Figure 2). Leg power was significantly correlated with both absolute V˙O_2_peak (*r* = 0.78; 95% CI: 0.62–0.88; *p* < 0.001) and relative V˙O_2_peak (*r* = 0.42; 95% CI: 0.12–0.65; *p* = 0.007), whereas leg fat-free mass was only significantly correlated with absolute V˙O_2_peak (*r* = 0.82; 95% CI: 0.68–0.90; *p* < 0.001) but not with relative V˙O_2_peak (*r* = 0.24; 95% CI: −0.07 to 0.50; *p* = 0.126).

## 4 Discussion

The present study provides an integrated evaluation of central hemodynamics, ventilatory efficiency, peripheral oxygen delivery and utilization, and skeletal muscle function in patients with HFpEF. The major new findings of this study are that measures of peripheral muscle function showed the strongest associations with exercise capacity after adjustment for age, sex, and body fat mass. Specifically, leg power showed the strongest positive association with relative V˙O_2_peak, whereas leg fat-free mass showed the strongest association with absolute V˙O_2_peak. Stroke volume and heart rate also showed positive associations, whereas V˙E/V˙CO_2_, SmO_2_, and tHbmass showed only trivial effects. Taken together, these findings indicate that peripheral muscle function was more strongly associated with exercise capacity than central hemodynamic limitation, ventilatory inefficiency, or peripheral oxygen delivery and utilization within this cohort.

Exercise capacity is governed by the integrated oxygen pathway (8, 40). Oxygen must be delivered by the central circulation, transported to skeletal muscle, diffused to mitochondria, and ultimately used for oxidative metabolism. Skeletal muscle performance therefore represents the downstream expression of this cascade. In this context, leg power integrates structural and metabolic determinants, including muscle mass, capillary density, mitochondrial content, and peripheral oxygen extraction. Its association with V˙O_2_peak therefore reflects the integrated peripheral capacity for oxygen diffusion and utilization, rather than an isolated measure of muscle strength.

In the present cohort, BMI was comparatively low [25.6 (4.3) kg/m^2^], whereas prior HFpEF studies typically reported BMI values between 30 and 34 kg/m^2^ (18–20). Consistent with this difference in body mass, relative V˙O_2_peak was higher in the present study [22.0 (5.1) mL/kg/min vs. around 16–18 mL/kg/min], while absolute V˙O_2_peak was largely comparable [1,565 (501) mL/min vs. around 1,380–1,600 mL/min]. Thus, the more favorable relative V˙O_2_peak primarily reflects lower body mass rather than true differences in absolute exercise capacity.

Within this context, previous studies have consistently demonstrated that skeletal muscle characteristics contribute to exercise intolerance in HFpEF. In cohorts with lower exercise capacity (approximately 1,180 mL/min or 14.5 mL/kg/min) (16, 17), reduced lean body mass and adverse muscle composition, particularly increased intermuscular fat, were closely associated with diminished V˙O_2_peak. In contrast, in HFpEF cohorts with exercise capacity comparable to the present study, sarcopenia has been linked to reduced functional performance and quality of life (18), and similar studies have emphasized impairments in muscle quality and function rather than muscle quantity (19, 41). Together, these findings suggest that muscle function and quality may be more relevant for relative exercise capacity than muscle quantity alone.

In line with these findings, leg power showed the strongest positive association with relative V˙O_2_peak, whereas leg fat-free mass showed the strongest association with absolute V˙O_2_peak. This pattern is consistent with the bivariate analyses, which demonstrated a moderate correlation between leg power and relative V˙O_2_peak, while the association with leg fat-free mass remained non-significant (*r* = 0.42; 95% CI: 0.12 to 0.65; *p* = 0.007 vs. *r* = 0.24; 95% CI: −0.07 to 0.50; *p* = 0.126). In contrast, leg fat-free mass showed a stronger association with absolute V˙O_2_peak, indicating that muscle quantity may contribute more to absolute oxygen uptake, but appears less relevant for relative exercise capacity.

Central hemodynamics (stroke volume and heart rate) showed positive associations in both models. Their similar contribution to relative and absolute V˙O_2_peak suggests that these central determinants of exercise capacity are relevant independent of body weight, but less strongly associated with V˙O_2_peak than peripheral muscle function. Prior studies have emphasized impaired stroke volume reserve, chronotropic incompetence, and elevated filling pressures as dominant abnormalities in HFpEF (42–44).

However, peak cardiac output in the present cohort reached 14.5 (3.4) L/min and was higher than values reported in previous studies, where cardiac output ranged from approximately 9–11 L/min (19, 42, 44). This relatively preserved capacity for oxygen delivery likely reduces the contribution of cardiac output to differences in V˙O_2_peak and shifts greater explanatory weight toward peripheral determinants. This interpretation is consistent with the relatively mild HFpEF phenotype of the present cohort, reflected by preserved functional status, comparatively high exercise capacity, and limited evidence of severe hemodynamic impairment. In such a clinically stable, non-anemic, non-obese cohort without overt decompensation, central limitations may define the syndrome but do not necessarily account for a substantial proportion of reduced exercise capacity once peripheral mechanisms are considered.

V˙E/V˙CO_2_, SmO_2_, and tHbmass all demonstrated trivial effect sizes (standardized *β* between −0.1 and 0.1), indicating that ventilatory efficiency and oxygen delivery are unlikely to be primary contributors to reduced exercise capacity in HFpEF. Naito et al. (22) observed lower V˙O_2_peak and ventilatory inefficiency in anemic patients with HFpEF. In contrast, mean hemoglobin was within the normal range [13.5 (1.3) g/dl], and although tHbmass—reflecting absolute oxygen transport capacity—was lower than previously reported in HFpEF [683 (205) g vs. 796 (166) g] (45), it was not associated with V˙O_2_peak. These findings suggest that ventilatory inefficiency and impaired oxygen transport may contribute to exercise intolerance primarily in the presence of anemia, whereas in non-anemic HFpEF, other mechanisms predominate.

While lower SmO_2_ is typically associated with greater peripheral extraction, SmO_2_ in the present study decreased only slightly from rest to peak exercise [62 (8)% at peak exercise], indicating a blunted peripheral oxygen extraction response. This pattern is consistent with impaired peripheral skeletal muscle diffusion capacity as described by Houstis et al. (8), who identified reduced peripheral oxygen diffusion as a major determinant of diminished V˙O_2_peak. As previously described (8, 46, 47), increasing cardiac output shortens capillary transit time, which may limit oxygen diffusion and thereby increase mixed venous oxygen content. Agostoni et al. (48) further suggested that excessive increases in cardiac output may exceed muscular extraction capacity, potentially due to suboptimal blood flow distribution during exercise. In this context, the near-stable SmO_2_ observed here is compatible with limited peripheral diffusion capacity rather than insufficient oxygen delivery. Importantly, cardiac output was only a moderate predictor in the present models, supporting the concept that reduced exercise capacity reflects an interaction of central and peripheral factors rather than isolated hemodynamic limitation. Accordingly, the observed associations between peripheral muscle function and V˙O_2_peak may reflect their relationship with multiple aspects of peripheral oxygen diffusion and utilization capacity.

The models explained 62% of the variance in relative and 81% in absolute V˙O_2_peak. The higher *R*^2^ in the absolute model likely reflects contributions of body size-related variables, including sex, stroke volume, tHbmass and leg power. The remaining unexplained variance may reflect additional central determinants not captured in the present analysis, as well as peripheral factors more directly represented by a-vDO_2_ (13). Although a-vDO_2_ could not be included as an independent predictor, its importance in HFpEF has been consistently demonstrated (12–14, 23), where it has been identified as a major determinant of exercise capacity, linking reduced oxygen extraction to impairments in capillary density, mitochondrial function, and skeletal muscle perfusion (12, 13, 23). These mechanisms provide a physiological framework linking impaired peripheral oxygen extraction and reduced functional muscle performance, which may help explain the observed association between peripheral muscle function and V˙O_2_peak in the present cohort.

Exercise training is recommended in HFpEF (4–6). The observed associations between peripheral muscle function and V˙O_2_peak support the integration of resistance training strategies targeting lower-extremity muscle function. In clinically stable HFpEF patients with relatively preserved central hemodynamic reserve, enhancing skeletal muscle performance may represent a particularly effective strategy to improve exercise capacity.

### 4.1 Limitations

Despite the relatively small sample size and a comparatively high number of predictors, extensive diagnostic procedures were applied to ensure model validity. These included tests for linearity, homoscedasticity, and normality of residuals, as well as multicollinearity checks and model selection based on the AIC. Nonetheless, the potential for overfitting and unstable coefficient estimates due to the combination of limited sample size and multiple predictors cannot be entirely excluded (49). Furthermore, the cross-sectional design precludes causal inference.

Stroke volume and cardiac output were estimated using non-invasive methods (bioimpedance cardiography and the Fick principle, respectively). While these techniques are suitable for group-level assessments and have shown adequate reproducibility in a previous study (30), their inherent measurement variability may have limited the explanatory power of the regression models. While resting echocardiographic parameters were available, exercise echocardiography was not performed. Therefore, exercise-induced hemodynamic abnormalities known to contribute to exercise intolerance in HFpEF were not assessed. Exercise-induced pulmonary congestion was also not assessed and may represent an additional contributor to exercise intolerance in HFpEF.

A limitation is the use of near-infrared spectroscopy, which does not allow complete separation of signals originating from skeletal muscle and overlying adipose tissue. Consequently, SmO_2_ reflects a composite tissue oxygenation signal rather than isolated muscle oxygen saturation.

In addition, a-vDO_2_ was calculated indirectly using the Fick principle and could not be included in the regression models as an independent variable. Direct measurement of a-vDO_2_ is recommended in future studies investigating central and peripheral determinants of exercise intolerance in HFpEF. HFpEF is a heterogeneous syndrome, and the present cohort represented a relatively mild phenotype with comparatively high exercise capacity and limited evidence of severe hemodynamic impairment. Finally, the present findings derive from a predominantly clinically stable HFpEF cohort with a mean BMI in the overweight rather than obese range and may not be generalizable to patients with more advanced stages of the syndrome, pronounced hemodynamic impairment, anemia, or obesity.

## 5 Conclusions

In this cross-sectional analysis of patients with HFpEF, measures of peripheral muscle function showed the strongest associations with exercise capacity. Specifically, leg power showed the strongest association with relative V˙O_2_peak, whereas leg fat-free mass showed the strongest association with absolute V˙O_2_peak. In both models, these associations exceeded those observed for central hemodynamic parameters, including stroke volume and heart rate. In contrast, V˙E/V˙CO_2_, SmO_2_, and tHbmass showed trivial associations with exercise capacity, indicating that ventilatory efficiency and peripheral oxygen delivery and utilization were not major contributors to reduced exercise capacity in this population. These findings support the growing recognition that peripheral muscle function is strongly associated with exercise capacity in HFpEF and may reflect underlying structural and metabolic alterations in skeletal muscle. Future studies should incorporate direct measurements of a-vDO_2_ to better characterize peripheral extraction deficits. Given the observed relevance of peripheral muscle function, exercise interventions combining endurance and resistance modalities may be particularly effective in improving exercise capacity in patients with HFpEF.

## Data Availability

The raw data supporting the conclusions of this article will be made available by the authors, without undue reservation.
