# Supplementary material for: Determinants of exercise capacity in heart failure with preserved ejection fraction: central hemodynamics, ventilatory efficiency, and peripheral muscle oxygen utilization and function
Source: Front Cardiovasc Med. 2026 Jul 15;13:1854913. doi: 10.3389/fcvm.2026.1854913 (PMC13415947; doi:10.3389/fcvm.2026.1854913)
Supplement: Supplementary file 1 [file Datasheet1.docx]

**Supplemental material**

[**Table S1:** Comparison of linear and nonlinear models 2](#_Toc233044806)

[**Figure S1**: Plots assessing model assumptions of linearity, homogeneity of variance, and normality of residuals 2](#_Toc233044807)

[**Table S2:** Multiple linear regression predicting relative V̇O_2_peak (mL/kg/min) 3](#_Toc233044808)

[**Table S3:** Multiple linear regression predicting absolute V̇O_2_peak (mL/min) 3](#_Toc233044809)

[**Table S4:** Sensitivity analysis: multiple linear regression predicting relative V̇O_2_peak (mL/kg/min) 4](#_Toc233044810)

[**Table S5:** Sensitivity analysis: multiple linear regression predicting absolute V̇O_2_peak (mL/min) 4](#_Toc233044811)

[**Figure S2**: Forest plot of standardized regression coefficients for determinants of absolute (grey) and relative (blue) V̇O_2_peak, based on original data, adjusted for age, sex, and body fat mass. 5](#_Toc233044812)

# **Table S1:** Comparison of linear and nonlinear models

| **Outcome** | **F-statistic** | **p-value** | **AIC (linear)** | **AIC (nonlinear)** | **ΔAIC** | **Preferred model** |
| --- | --- | --- | --- | --- | --- | --- |
| Absolute V̇O₂peak | 2.32 | 0.125 | 402 | 517 | 115 | Linear |
| Relative V̇O₂peak | 2.97 | 0.070 | 169 | 279 | 110 | Linear |

For both absolute and relative V̇O₂peak, model comparisons revealed no significant difference between linear and nonlinear models. V̇O₂peak: peak oxygen uptake, AIC: Akaike Information Criterion


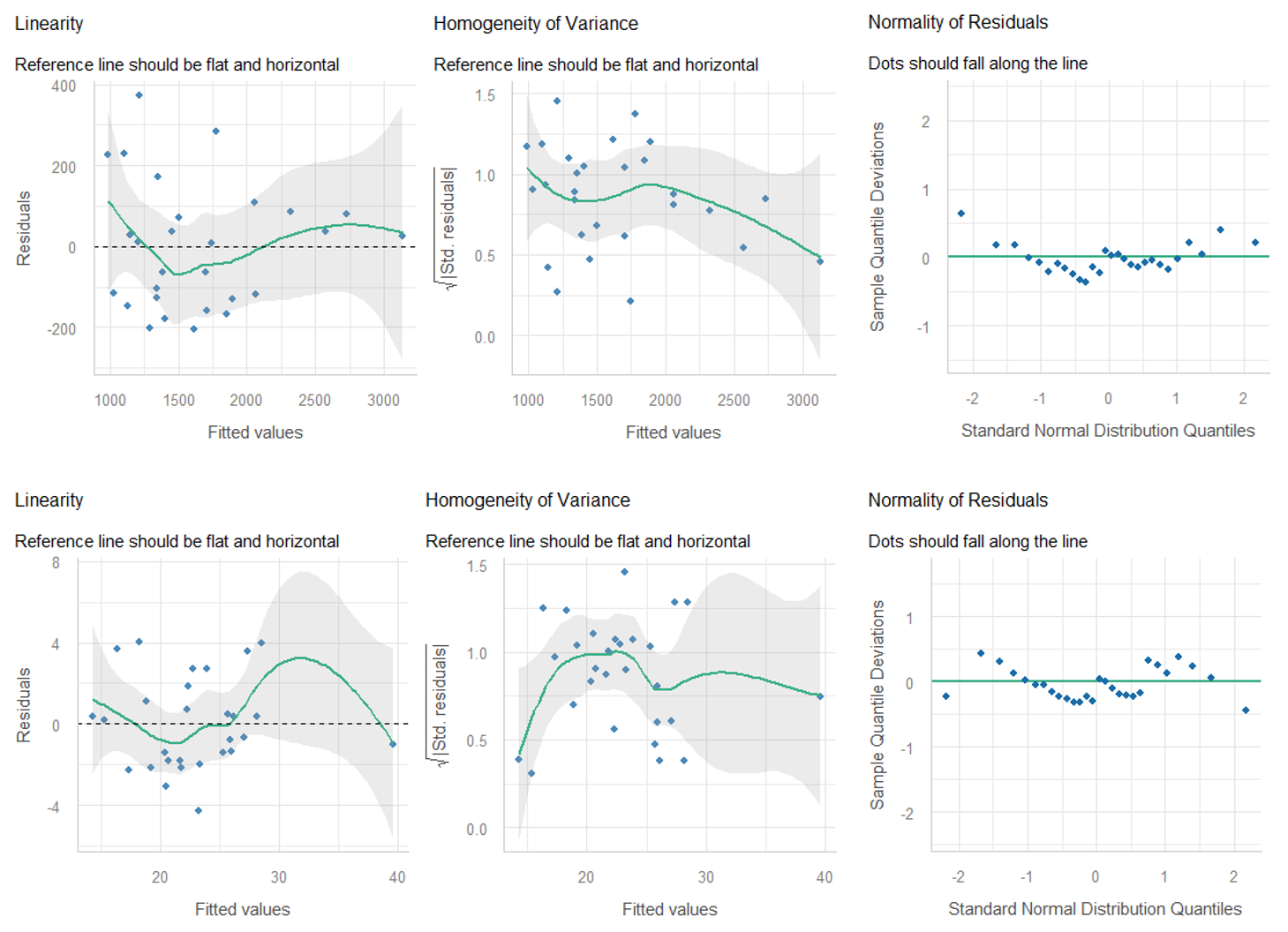


**Figure S1**: Plots assessing model assumptions of linearity, homogeneity of variance, and normality of residuals. Upper models: absolute V̇O₂peak (mL/min); lower models: relative V̇O₂peak (mL/kg/min).

Table S2 shows the unstandardized regression coefficients for the model predicting relative V̇O_2_peak.

# **Table S2:** Multiple linear regression predicting relative V̇O_2_peak (mL/kg/min)

| **Predictor** | **β (unstd.)** | **SE** | **95% CI** | **p value** |
| --- | --- | --- | --- | --- |
| (Intercept) | 4.42 | 15.95 | (-28.35; 37.2) | 0.784 |
| Body fat mass (kg) | -0.39 | 0.09 | (-0.57; -0.21) | < 0.001 |
| Stroke volume (mL) | 0.06 | 0.03 | (0.00; 0.12) | 0.054 |
| Heart rate (bpm) | 0.07 | 0.04 | (-0.01; 0.15) | 0.097 |
| Leg Power (kW) | 2.57 | 1.95 | (-1.44; 6.58) | 0.200 |
| Fat free mass legs (kg) | 0.31 | 0.35 | (-0.40; 1.02) | 0.379 |
| V̇E/V̇CO_2_ | 0.06 | 0.12 | (-0.18; 0.30) | 0.601 |
| SmO_2_ | 0.04 | 0.09 | (-0.15; 0.23) | 0.682 |
| Age | -0.04 | 0.12 | (-0.29; 0.21) | 0.754 |
| Sex (male) | -0.58 | 2.62 | (-5.97; 4.81) | 0.826 |
| Total hemoglobin mass (g) | 0.00 | 0.01 | (-0.01; 0.01) | 0.884 |

V̇O_2_peak: peak oxygen uptake, β (unstd.): unstandardized regression coefficient, SE: standard error; 95% CI: 95% confidence interval, SmO_2_: muscle oxygen saturation, V̇E/V̇CO_2_: ratio of minute ventilation to carbon dioxide output slope

Model statistics: R^2^ = 0.62 (95% CI 0.40-0.78), adjusted R^2^ = 0.50, F (10, 32) = 5.3, p < 0.001, n = 43.

Table S3 shows the unstandardized regression coefficients for the model predicting absolute V̇O_2_peak.

# **Table S3:** Multiple linear regression predicting absolute V̇O_2_peak (mL/min)

| **Predictor** | **β (unstd.)** | **SE** | **95% CI** | **p value** |
| --- | --- | --- | --- | --- |
| (Intercept) | -1127.08 | 1105.49 | (-3397.84; 1143.68) | 0.986 |
| Fat free mass legs (kg) | 79.76 | 24.30 | (30.10; 129.42) | 0.003 |
| Stroke volume (mL) | 4.32 | 2.12 | (-0.01; 8.64) | 0.050 |
| Heart rate (bpm) | 5.20 | 2.86 | (-0.66; 11.06) | 0.080 |
| Leg Power (kW) | 210.94 | 134.92 | (-65.87; 487.75) | 0.130 |
| Body fat mass (kg) | -5.42 | 6.15 | (-18.02; 7.18) | 0.385 |
| V̇E/V̇CO_2_ | 3.97 | 8.08 | (-12.56; 20.50) | 0.627 |
| Total hemoglobin mass (g) | 0.06 | 0.37 | (-0.71; 0.83) | 0.873 |
| Age | -1.38 | 8.57 | (-18.94; 16.18) | 0.886 |
| SmO_2_ | 0.30 | 6.64 | (-13.32; 13.93) | 0.965 |
| Sex (male) | 3.28 | 182.42 | (-371.24; 377.80) | 0.986 |

V̇O_2_peak: peak oxygen uptake, β (unstd.): unstandardized regression coefficient, SE: standard error; 95% CI: 95% confidence interval, SmO_2_: muscle oxygen saturation, V̇E/V̇CO_2_: ratio of minute ventilation to carbon dioxide output slope

Model statistics: R^2^ = 0.81 (95% CI 0.67-0.89), adjusted R^2^ = 0.75, F (10, 32) = 13.3, p < 0.001, n = 43.

Table S4 and S5 show the standardized regression coefficients for sensitivity analyses predicting relative and absolute V̇O_2_peak, respectively, after exclusion of patients with NYHA class I.

# **Table S4:** Sensitivity analysis: multiple linear regression predicting relative V̇O_2_peak (mL/kg/min)

| **Predictor** | **β (std.)** | **SE** | **95% CI** | **p value** | **LMG** |
| --- | --- | --- | --- | --- | --- |
| (Intercept) | -0.46 | 0.85 | (-2.25; 1.34) | 0.597 | – |
| Body fat mass (kg) | -0.60 | 0.21 | (-1.04; -0.17) | 0.010 | 36.9% |
| Stroke volume (mL) | 0.26 | 0.15 | (-0.06; 0.58) | 0.104 | 10.3% |
| Heart rate (bpm) | 0.27 | 0.20 | (-0.16; 0.70) | 0.200 | 10.7% |
| Leg Power (kW) | 0.30 | 0.35 | (-0.44; 1.05) | 0.400 | 12.3% |
| Age | -0.20 | 0.29 | (-0.80; 0.41) | 0.501 | 10.7% |
| SmO_2_ | 0.13 | 0.19 | (-0.28; 0.54) | 0.504 | 2.7% |
| Sex (male) | 0.35 | 0.64 | (-0.99; 1.69) | 0.593 | 6.3% |
| Leg fat-free mass (kg) | -0.19 | 0.36 | (-0.94; 0.57) | 0.608 | 4.7% |
| Total hemoglobin mass (g) | 0.08 | 0.27 | (-0.52; 0.67) | 0.780 | 2.2% |
| V̇E/V̇CO_2_ | 0.04 | 0.19 | (-0.37; 0.45) | 0.823 | 3.1% |

LMG values indicate the relative contribution of each predictor to the explained variance (R^2^) of the model

V̇O_2_peak: peak oxygen uptake, β (std.): standardized regression coefficient, SE: standard error; 95% CI: 95% confidence interval, LMG: Lindeman-Merenda-Gold, SmO_2_: muscle oxygen saturation, V̇E/V̇CO_2_: ratio of minute ventilation to carbon dioxide output slope

Model statistics: R^2^ = 0.66 (95% CI 0.40-0.82), adjusted R^2^ = 0.48, F (10, 20) = 3.8, p = 0.005, n = 31.

# **Table S5:** Sensitivity analysis: multiple linear regression predicting absolute V̇O_2_peak (mL/min)

| **Predictor** | **β (std.)** | **SE** | **95% CI** | **p value** | **LMG** |
| --- | --- | --- | --- | --- | --- |
| (Intercept) | -0.67 | 0.71 | (-2.17; 0.83) | 0.360 | – |
| Stroke volume (mL) | 0.20 | 0.13 | (-0.06; 0.47) | 0.124 | 5.7% |
| Heart rate (bpm) | 0.25 | 0.17 | (-0.10; 0.61) | 0.152 | 11.7% |
| Leg Power (kW) | 0.31 | 0.29 | (-0.30; 0.91) | 0.295 | 25.3% |
| Sex (male) | 0.51 | 0.53 | (-0.61; 1.63) | 0.354 | 14.4% |
| Age | -0.16 | 0.24 | (-0.66; 0.34) | 0.517 | 15.5% |
| Leg fat-free mass (kg) | 0.17 | 0.30 | (-0.46; 0.80) | 0.572 | 19.3% |
| Body fat mass (kg) | -0.07 | 0.17 | (-0.43; 0.30) | 0.705 | 0.9% |
| SmO_2_ | 0.05 | 0.16 | (-0.29; 0.39) | 0.759 | 1.2% |
| Total hemoglobin mass (g) | 0.03 | 0.22 | (-0.45; 0.50) | 0.905 | 4.8% |
| V̇E/V̇CO_2_ | 0.01 | 0.16 | (-0.32; 0.35) | 0.931 | 1.1% |

LMG values indicate the relative contribution of each predictor to the explained variance (R^2^) of the model

V̇O_2_peak: peak oxygen uptake, β (std.): standardized regression coefficient, SE: standard error; 95% CI: 95% confidence interval, LMG: Lindeman-Merenda-Gold, SmO_2_: muscle oxygen saturation, V̇E/V̇CO_2_: ratio of minute ventilation to carbon dioxide output slope

Model statistics: R^2^ = 0.76 (95% CI 0.55-0.88), adjusted R^2^ = 0.64, F (10, 20) = 6.2, p < 0.001, n = 31

Figure S2 presents the forest plot based on the original dataset (non-imputed) to illustrate robustness of associations.


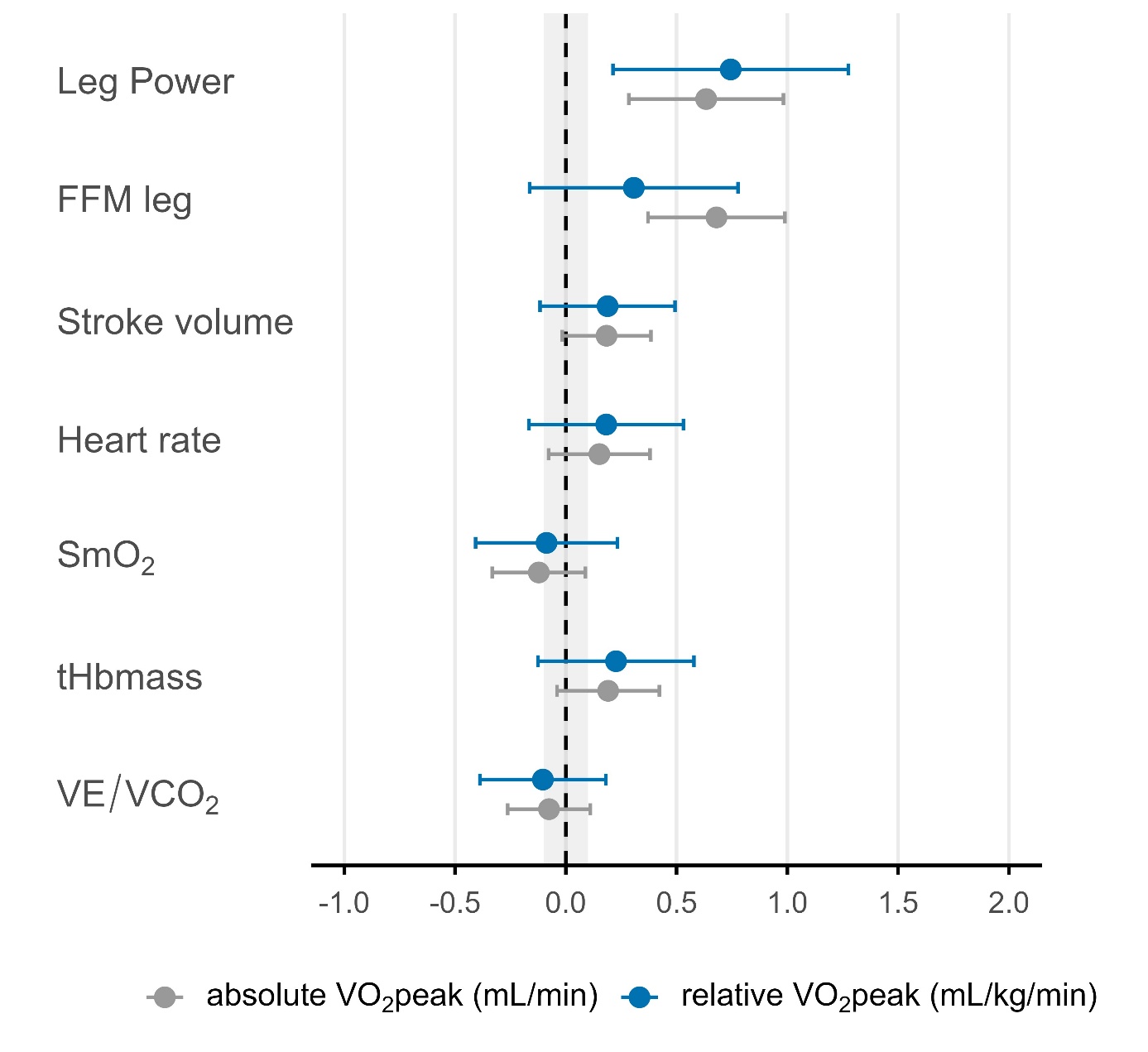


**Figure S2**: Forest plot of standardized regression coefficients for determinants of absolute (grey) and relative (blue) V̇O_2_peak, based on original data, adjusted for age, sex, and body fat mass. Points represent mean standardized regression coefficients; horizontal bars indicate 95% confidence intervals. The grey-shaded area (-0.1 to 0.1) denotes a range interpreted as a trivial effect size.

FFM leg: leg fat-free mass, SmO_2_: muscle oxygen saturation, tHbmass: total hemoglobin mass, V̇E/V̇CO_2_: ratio of minute ventilation to carbon dioxide output slope, V̇O_2_peak: peak oxygen uptake, BMI: body mass index.
